# Supplementary material for: Epigenetic characterization of housekeeping core promoters and their importance in tumor suppression
Source: Nucleic Acids Res. 2023 Dec 12;52(3):1107–19. doi: 10.1093/nar/gkad1164 (PMC10853790; doi:10.1093/nar/gkad1164)
Supplement: gkad1164_supplemental_files [file gkad1164_supplemental_files.zip › Supplementary_notes.docx]

**Supplementary note 1. Detailed analysis on the absence of certain HKGs in Figure 3A**

In Figure 3A, it is noted that 112 housekeeping genes (HKGs) are not included in the categorization of HKGs.

The omission of certain core promoters of HKGs in Figure 3A can be largely attributed to challenges associated with the pre-processing of cis-regulatory elements (CREs). More specifically, to the merging and unsupervised annotation processes:

- Merging process: Core promoter regions of HKGs might not be identified as housekeeping CREs. In other words, they might be identified as CREs active in less than 45 cell types (the definition of housekeeping elements used in this project).

- Unsupervised annotation process: Core promoter regions of HKGs might be annotated as a different class. For example, they might be annotated as promoters, enhancers, etc.

To further illustrate these inherent and potentially unavoidable technical biases, we conducted the following supplementary analyses:

- For the whole set of CREs (e.g., HK-CREs, cell type-specific CREs, etc.), we compiled a list of housekeeping genes (HKGs) whose transcription start sites (TSS) fall within a 300-base pair window centered on each CRE (the same window width employed in our manuscript). We then compared this list with the set of 2,131 HKGs. We observed an increase in the number of identified HKGs, from 2,019 genes (94.7% of all HKGs, as shown in Figure 3A) to 2,109 genes (98.9% of all HKGs). These numbers continued to rise with wider windows. For instance, employing a 1-kilobase pair window allowed us to capture 2,123 HKGs, corresponding to approximately 99.6% of the entire set of HKGs.
- We further explored the later analysis employing a 1-kilobase pair window (refer to the subsequent two Figures). For each HKG, we identified the nearest CREs to any of its TSSs. Although most of the HKGs were associated with HK-CREs, some other CREs were found in proximity to HKGs, as depicted in the top plot. We noticed a small peak in the left-bottom part of the plot suggesting a relatively high number of CREs associated with a low “number of cell types”, e.g., cell type-specific CREs. We attribute this small peak to the high number of CREs from a low “number of cell types” (see Figure 1A in the main manuscript). To correct this inherent bias we normalized the number of CREs associated with HKGs by dividing them by the total number of CREs found on each “number of cell types”, e.g., the numbers in Figure 1A of the main manuscript. The bottom plot presents the calculated proportions. The small peak was corrected and the proportion of CREs associated with HKGs shows a rising trend towards a higher “number of cell types”. This confirms that, despite some technical biases in the annotation of HK-CREs, they generally exhibit a stronger association with HKGs than other CREs from a lower “number of cell types”.


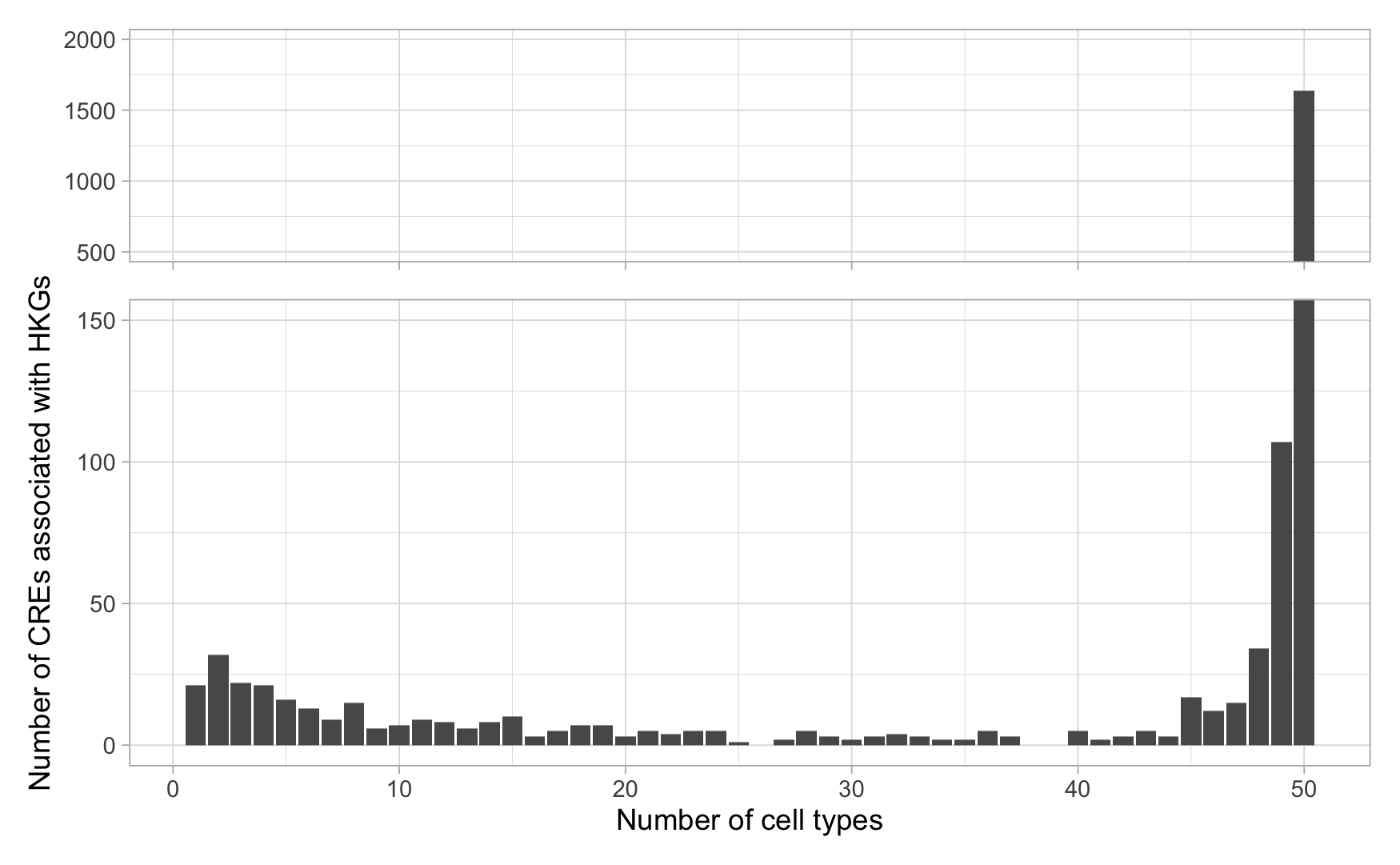

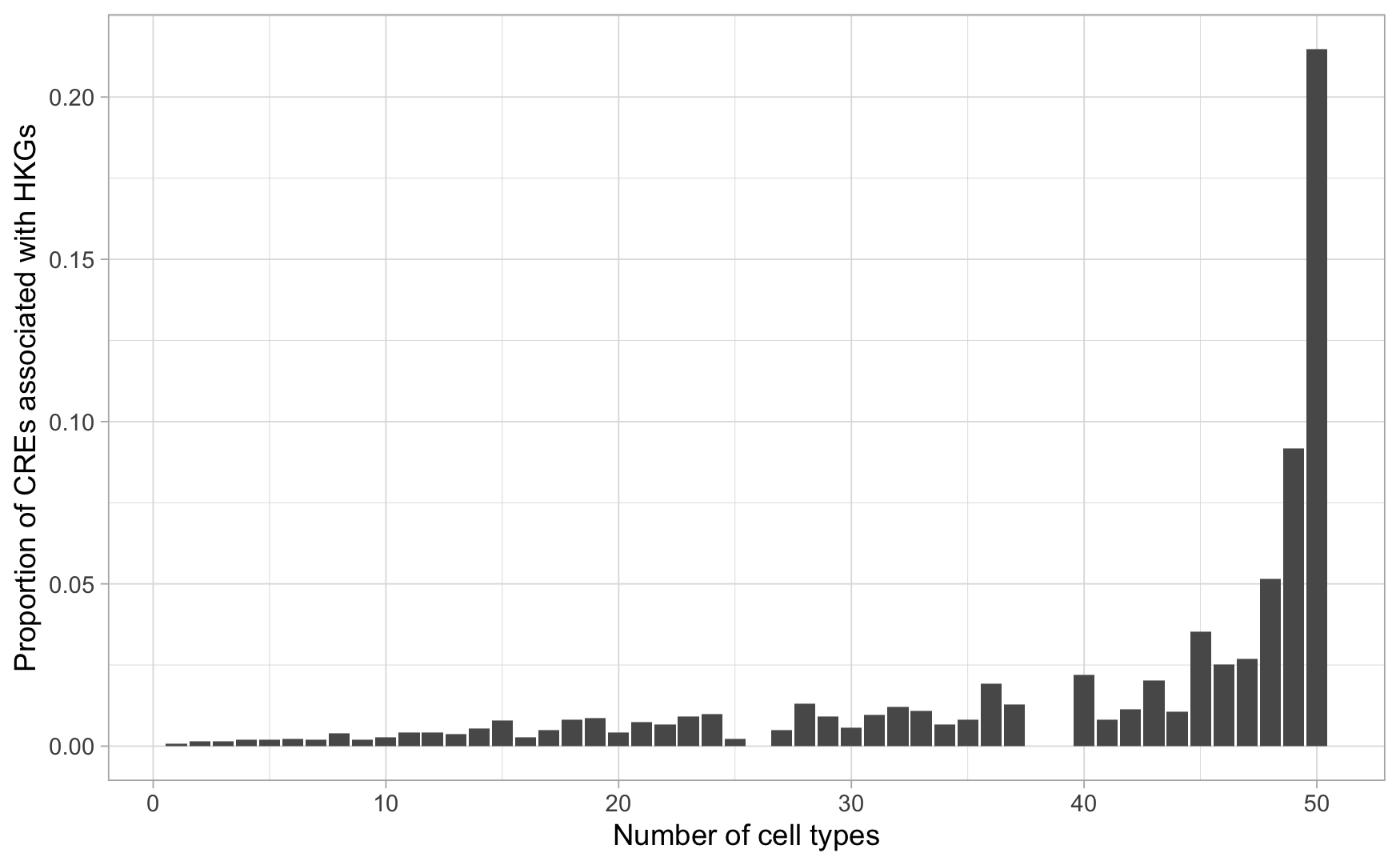


Small peak

- To illustrate the possible technical biases during the unsupervised annotation of HK-CREs, we obtained the list of housekeeping genes (HKGs) whose transcription start sites (TSS) are within 300 base pairs of the center of any housekeeping cis-regulatory elements (HK-CREs), such as core promoters and promoters. We observed an increase in the number of HKGs identified, from 2,019 genes (94.7% of all HKGs, as shown in Figure 3A of the manuscript) to 2,050 genes (96.1% of all HKGs). The following table summarizes the labels for HK-CREs associated with HKGs. We discovered that only HK-CREs labeled as core promoters, promoters, enhancer short distance, and H3K4me3 negative were connected to HKGs. On the other hand, we didn’t find an association between “other enhancer long” and “super long distance”, nor with other CREs with low levels of histone marks. This demonstrates that, although not flawless, our labeling effectively captures a comprehensive overview of the intrinsic relationship between HK-CREs labeled as core promoters and the collection of HKGs.

| Label | Number of HK-CREs associated with a HKG |
| --- | --- |
| Core promoters (CP) | 2,151 |
| Promoters (P) | 16 |
| Enhancer short distance (ESD) | 12 |
| Enhancer long distance (ELD) | 0 |
| Enhancer super long distance (ESLD) | 0 |
| Enhancer short distance inactive (ESDi) | 0 |
| Other medium distance (OMD) | 0 |
| Other long distance (OLD) | 0 |
| H3K4me1 negative | 22 |

Please note that the sum of the number of HK-CREs linked to an HKG is greater than the number of HKGs linked to HK-CREs. This discrepancy occurs because a single HKG can be associated with multiple HK-CREs.
